# White adipose tissue reference network: a knowledge resource for exploring health-relevant relations

**DOI:** 10.1007/s12263-014-0439-x

**Published:** 2014-12-03

**Authors:** Thomas Kelder, Georg Summer, Martien Caspers, Evert M. van Schothorst, Jaap Keijer, Loes Duivenvoorde, Susanne Klaus, Anja Voigt, Laura Bohnert, Catalina Pico, Andreu Palou, M. Luisa Bonet, Aldona Dembinska-Kiec, Malgorzata Malczewska-Malec, Beata Kieć-Wilk, Josep M. del Bas, Antoni Caimari, Lluis Arola, Marjan van Erk, Ben van Ommen, Marijana Radonjic

**Affiliations:** 1 Microbiology & Systems Biology, TNO, Zeist, The Netherlands; 2 CARIM, Maastricht University, Maastricht, The Netherlands; 3 Human and Animal Physiology, Wageningen University, Wageningen, The Netherlands; 4 Group of Energy Metabolism, German Institute of Human Nutrition in Potsdam, Nuthetal, Germany; 5 Molecular Biology, Nutrition and Biotechnology (Nutrigenomics), University of the Balearic Islands (UIB), Palma de Mallorca, Spain; 6 CIBER Fisiopatología de la Obesidad y Nutrición (CIBEROBN), Palma de Mallorca, Spain; 7 Department of Clinical Biochemistry, Jagiellonian University Medical College, Krakow, Poland; 8 Department of Metabolic Disorders, Jagiellonian University Medical College, Krakow, Poland; 9 Centre Tecnològic de Nutrició i Salut (CTNS), TECNIO, Reus, Spain; 10 Rovira i Virgili University, Tarragona, Spain; 11 Present Address: EdgeLeap B.V., Hooghiemstraplein 15, 3514 AX Utrecht, The Netherlands

**Keywords:** Network biology, Systems biology, Data integration, Adipose tissue, Nutrition, Drugs

## Abstract

Optimal health is maintained by interaction of multiple intrinsic and environmental factors at different levels of complexity—from molecular, to physiological, to social. Understanding and quantification of these interactions will aid design of successful health interventions. We introduce the reference network concept as a platform for multi-level exploration of biological relations relevant for metabolic health, by integration and mining of biological interactions derived from public resources and context-specific experimental data. A White Adipose Tissue Health Reference Network (WATRefNet) was constructed as a resource for discovery and prioritization of mechanism-based biomarkers for white adipose tissue (WAT) health status and the effect of food and drug compounds on WAT health status. The WATRefNet (6,797 nodes and 32,171 edges) is based on (1) experimental data obtained from 10 studies addressing different adiposity states, (2) seven public knowledge bases of molecular interactions, (3) expert’s definitions of five physiologically relevant processes key to WAT health, namely WAT expandability, Oxidative capacity, Metabolic state, Oxidative stress and Tissue inflammation, and (4) a collection of relevant biomarkers of these processes identified by BIOCLAIMS (http://bioclaims.uib.es). The WATRefNet comprehends multiple layers of biological complexity as it contains various types of nodes and edges that represent different biological levels and interactions. We have validated the reference network by showing overrepresentation with anti-obesity drug targets, pathology-associated genes and differentially expressed genes from an external disease model dataset. The resulting network has been used to extract subnetworks specific to the above-mentioned expert-defined physiological processes. Each of these process-specific signatures represents a mechanistically supported composite biomarker for assessing and quantifying the effect of interventions on a physiological aspect that determines WAT health status. Following this principle, five anti-diabetic drug interventions and one diet intervention were scored for the match of their expression signature to the five biomarker signatures derived from the WATRefNet. This confirmed previous observations of successful intervention by dietary lifestyle and revealed WAT-specific effects of drug interventions. The WATRefNet represents a sustainable knowledge resource for extraction of relevant relationships such as mechanisms of action, nutrient intervention targets and biomarkers and for assessment of health effects for support of health claims made on food products.

**Electronic supplementary material:**

The online version of this article (doi:10.1007/s12263-014-0439-x) contains supplementary material, which is available to authorized users.

## Introduction

Health is maintained by interplay among multiple intrinsic and environmental factors, which are interacting at different complexity levels. For instance, organ functioning is determined by multiple (psycho)physiological processes, which can in turn be modified by chemical compounds that act via molecular networks of genes, proteins or lipid species (Oltvai and Barabási 2002; Schadt and Björkegren 2012; Barabási et al. 2011). To achieve and maintain optimal health, it is important to understand such complex biological relations—from molecular, to physiological, to social—and to determine elements and paths whose modification will drive system toward a desired state (Barabási 2007). This calls for approaches that can comprehend complex relations and account for multiple biological levels in order to build a coherent picture (“a signature”) of health.

A quest for defining and quantifying health status and effects of interventions to improve health is facilitated by technological advances in the last decades. By expansion of high-throughput screening methods, next-generation sequencing technology, self-monitoring devices, online information-sharing platforms and similar technological breakthroughs, we have now acquired the means to measure, share, and compute (personal) data and knowledge at an increasingly large scale (Chen et al. 2012; Murdoch and Detsky 2013; Field et al. 2009). Network-based methods provide a platform to integrate and organize such diverse and abundant (“big”) data into a knowledge resource by bridging multiple data silos at multiple biological levels (Barabási and Oltvai 2004). For instance, biological networks can be built to integrate experimental data with prior knowledge about molecular interactions (protein–protein, protein–DNA and ligand–receptor), regulatory aspects (transcription factor, miRNA targets and epigenetics), mechanistic context (signalling cascades and metabolic pathways), tissue specificity, association of molecular phenotypes and processes with (patho)physiological conditions, outcomes of self-assessment questionnaires, etc. Such comprehensive relational maps can be mined using network-based algorithms for associations with specific health and disease aspects (Langfelder and Horvath 2008; Carter et al. 2013) and for extraction of features of interest, such as key submodules (Mitra et al. 2013) and regulators, intervention targets and biomarkers (Hofree et al. 2013; Dudley and Butte 2009; Wang and Chen 2011). It is becoming evident that this approach facilitates discovery of more robust biomarkers and intervention targets compared to solely correlation-based feature selection methods (Roukos 2010), namely it allows identification of features whose mechanistic context implies their key role in physiologically relevant processes, which in turn drive the functioning of (systems of) organs, consolidating the cause–effect relationship between molecular changes and a health effect.

Approaches that include all relevant parameters and interactions of the biological system are particularly relevant in deciphering metabolic health and disease, as imbalance in metabolic homeostasis can be triggered by multiple, heterogeneous and often subtle intrinsic and environmental changes (Corthésy-Theulaz et al. 2005). To maintain metabolic health, many organs and systems need to function synchronously and within their optimal functioning range. Important metabolic health determinants include: liver substrate handling, white adipose tissue energy storage capacity, pancreatic insulin production, muscle metabolic response to exercise, vasculature hemodynamics and adequate immune response (Leviston 2011). The complete metabolic system strives to maintain homeostasis in continuously changing metabolic conditions. Yet, this comes at a cost during chronic metabolic stress, inducing adaptive mechanisms that may lead to pathologies. Until the resilience limits of these systems are reached, metabolic imbalance can be largely reversed, often by appropriate lifestyle intervention such as diet and/or exercise (Radonjic et al. 2013; Van Ommen et al. 2014). Nevertheless, the compliance to lifestyle interventions is a major problem, and it is not equally effective for all persons (Fappa et al. 2008). Therefore, understanding the molecular network controlling processes required for the maintenance of metabolic health and effects of interventions on this network is of interest for designing effective lifestyle intervention programs tailored to fit person-specific (psycho)physiological makeup, as well as for the development of drugs that will mimic broad systems efficacy and minimal adverse effects of lifestyle interventions.

The case of adipose tissue is particularly interesting as it is constituted by different depots distributed in different parts of the body, the so-called adipose organ (Cinti 2005). What matters is not only the fat but where it is in the body, what type of fat and nonfat cells complement the depot and the notion that healthy fat is when functionality to accumulate fat is working to protects the body by providing a “safe home” for lipids, which can be toxic to other tissues such as muscle or the liver (Owens 2014). The inappropriate accumulation of lipids in fat depots and, consequently, in tissues that are not equipped to handle them results in continued low-grade inflammation and, ultimately, in metabolic disease, insulin resistance and type 2 diabetes (Owens 2014).

To facilitate multi-level exploration of biological relations relevant for metabolic health, we introduce the reference network concept as a platform for integration and mining of biological interactions derived from public resources and context-specific experimental data. Within the FP7 BIOCLAIMS project (http://bioclaims.uib.es/), which focuses on discovery of biomarkers for assessing the benefits of health-promoting food compounds, we have built a reference network using white adipose tissue (WAT) health as an endpoint of interest. The White Adipose Tissue Health Reference Network (WATRefNet) is based on (1) experimental data obtained from 10 studies addressing different adiposity states, (2) seven public knowledge bases of molecular interactions, (3) expert’s definitions of physiologically relevant processes key to WAT health and (4) collection of relevant biomarkers of these processes identified by BIOCLAIMS. The WATRefNet comprehends multiple layers of biological complexity as it contains various types of nodes and edges that represent different biological levels (genes, clinical chemistry and physiological parameters measured in either WAT, peripheral blood mononuclear cells (PBMCs), or blood) and interactions (protein–protein interactions, protein–metabolite interactions, transcription factor targets, microRNA targets, pathway interactions and protein–drug interactions). The resulting network has been used to extract subnetworks specific to physiological processes key to WAT health, namely WAT expandability, Oxidative capacity, Metabolic state, Oxidative stress and Tissue inflammation. Each of these signatures represents a mechanistically sustained composite biomarker for assessment and quantification of the effect of interventions on a physiological aspect that determines WAT health status. In addition, the WATRefNet is currently being (re)used in associated projects (Bobeldijk et al. 2014) as a knowledge resource for extraction of relevant relationships such as mechanisms of action, nutrient intervention targets and assessment of health status.

## Results

### Definition of physiological processes determining WAT health

To set a framework for building a reference network of biological relations relevant for WAT health, the FP7 BIOCLAIMS consortium identified five most relevant physiological processes determining WAT health status: WAT (referred to as “Adipose”) expandability (Slawik and Vidal-Puig 2007), Oxidative capacity (De Pauw et al. 2009), Metabolic state (Klaus 2004), Oxidative stress (Furukawa et al. 2004) and Tissue inflammation (Wellen and Hotamisligil 2003). Subsequently, for each of these processes, biomarkers were assigned, defined as a known assay readout parameters that best represent or are associated with the given physiological process. These expert-defined biomarkers were further used as “anchor nodes” for connecting molecular part of the reference network to the physiological endpoints determining WAT health. The list of expert’s knowledge-based processes and associated markers is provided in Table 1.

**Table 1:** Expert’s knowledge-based processes and associated markers as defined by the BIOCLAIMS consortium including main results from the integrated network analysis

Name	Process	Tissue	Nr. studies measured	Significant across studies	Consistent fold-change	Nr. Seed node neighbors
Adipocyte area	Adipose expandability	Adipose	0	–	–	0
Adiponectin	Adipose expandability	Blood	0	–	–	1
Adipose mass MRI	Adipose expandability		0	–	–	0
**Adipose tissue mass**	**Adipose expandability**		**6**	**Yes**	**Yes**	**6**
**Brown fat mass**	**Adipose expandability**		**3**	**Yes**	**Yes**	**5**
**Epididymal adipose mass**	**Adipose expandability**		**5**	**Yes**	**Yes**	**3**
Leptin	Adipose expandability	Blood	0	–	–	5
**Leptin Adiponectin ratio**	**Adipose expandability**	**Blood**	**5**	**Yes**	**Yes**	**2**
**MEST1**	**Adipose expandability**	**Adipose**	**4**	**Yes**	**Yes**	**0**
Subcutaneous adipose mass^#^	Adipose expandability		1	No	No	0
Visceral adipose mass	Adipose expandability		4	No	Yes	12
Acadvl^#^	Metabolic state	Adipose	7	No	Yes	1
**Acc**	**Metabolic state**	**Adipose**	**7**	**Yes**	**Yes**	**2**
Acyl carnitines	Metabolic state	Blood	0	–	–	0
Adiponectin	Metabolic state	Adipose	7	No	No	6
ATGL	Metabolic state	Adipose	7	No	No	7
BCAA	Metabolic state	Blood	0	–	–	0
CPT1 (PBMC)^#^	Metabolic state	PBMC	2	Yes	No	0
**CPT1a**	**Metabolic state**	**Adipose**	**7**	**Yes**	**Yes**	**5**
Cpt1b	Metabolic state	Adipose	6	No	No	5
Dgat2^#^	Metabolic state	Adipose	7	No	No	1
FABP4	Metabolic state	Adipose	7	No	Yes	3
FABPpm^#^	Metabolic state	Adipose	7	No	No	1
FAS	Metabolic state	Adipose	7	No	No	9
**FASN**	**Metabolic state**	**Adipose**	**7**	**Yes**	**Yes**	**7**
**FASN (PBMC)**	**Metabolic state**	**PBMC**	**2**	**Yes**	**Yes**	**0**
**GLUT4**	**Metabolic state**	**Adipose**	**7**	**Yes**	**Yes**	**6**
Gpat	Metabolic state	Adipose	7	No	No	3
GyK^#^	Metabolic state	Adipose	7	No	No	0
Hsl	Metabolic state	Adipose	7	No	No	5
HSL (PBMC)^#^	Metabolic state	PBMC	1	No	Yes	0
INSR	Metabolic state	Adipose	7	No	No	9
IRS1	Metabolic state	Adipose	7	No	No	15
Lactate	Metabolic state	Blood	0	–	–	0
**LDHa**	**Metabolic state**	**Adipose**	**4**	**Yes**	**Yes**	**4**
LepR	Metabolic state	Adipose	7	No	No	3
**Lpl**	**Metabolic state**	**Adipose**	**7**	**Yes**	**Yes**	**10**
Lysophosphatidylinositols (plasma)	Metabolic state	Blood	0	–	–	0
**PDK**	**Metabolic state**	**Adipose**	**7**	**Yes**	**Yes**	**3**
**PFK**	**Metabolic state**	**Adipose**	**7**	**Yes**	**Yes**	**0**
PGC1a	Metabolic state	Adipose	7	No	Yes	7
**PKM**	**Metabolic state**	**Adipose**	**4**	**Yes**	**Yes**	**6**
Ppara	Metabolic state	Adipose	7	No	No	16
Resistin	Metabolic state	Blood	0	–	–	1
**RXRB (PBMC)**	**Metabolic state**	**PBMC**	**2**	**Yes**	**Yes**	**0**
SIRT1	Metabolic state	Adipose	4	No	No	26
Tyrosine hydroxylase level^#^	Metabolic state	Adipose	6	No	No	1
**UCP2**	**Metabolic state**	**Adipose**	**7**	**Yes**	**Yes**	**3**
Visfatin	Metabolic state	Blood	0	–	–	0
Mito density (cardiolipin)	Oxidative capacity	Adipose	0	–	–	0
Mito density (citrate synthase level)	Oxidative capacity	Adipose	0	–	–	0
Mito density (EM)	Oxidative capacity	Adipose	0	–	–	0
**Mito density (mt/nDNA)**	**Oxidative capacity**	**Adipose**	**2**	**Yes**	**Yes**	**2**
Uncoupled oxygen consumption	Oxidative capacity	Adipose	0	–	–	0
Aconitase/citrate synthase activity	Oxidative stress	Adipose	0	–	–	0
**SOD1**	**Oxidative stress**	**Adipose**	**7**	**Yes**	**Yes**	**8**
SOD2	Oxidative stress	Adipose	7	Yes	No	7
TRXRD2	Oxidative stress	Adipose	0	–	–	0
**Adam8**	**Tissue inflammation**	**Adipose**	**7**	**Yes**	**Yes**	**0**
**Cd11c**	**Tissue inflammation**	**Adipose**	**4**	**Yes**	**Yes**	**2**
Cd163	Tissue inflammation	Adipose	7	No	No	3
Glut1	Tissue inflammation	Adipose	7	No	No	5
Gpx1	Tissue inflammation	Adipose	0	–	–	2
Hif1a	Tissue inflammation	Adipose	7	No	No	38
Il10	Tissue inflammation	Adipose	4	No	No	21
Il1b	Tissue inflammation	Adipose	0	–	–	0
Il6	Tissue inflammation	Adipose	4	No	No	50
Mgl2	Tissue inflammation	Adipose	0	–	–	0
**Mrc1**	**Tissue inflammation**	**Adipose**	**7**	**Yes**	**Yes**	**1**
Nos2	Tissue inflammation	Adipose	3	No	No	11
Ppargc1b^#^	Tissue inflammation	Adipose	5	No	No	1
Stat6	Tissue inflammation	Adipose	7	No	No	5
**Tnf**	**Tissue inflammation**	**Adipose**	**4**	**Yes**	**Yes**	**48**
Vegfa	Tissue inflammation	Adipose	7	No	Yes	48
Vhl	Tissue inflammation	Adipose	7	No	Yes	28

Expert-defined markers with consistent fold-change sign and aggregated FDR corrected *p* < 0.01 are marked in bold. Expert-defined markers which are measured but do not show either consistent change in the data or are in known molecular neighborhood of the seed nodes (nr. of seed neighbors >1) are marked with a hash

### Context-specific experimental data for the WAT health reference network

Publicly available experimental data and data from proprietary FP7 BIOCLAIMS studies addressing different adiposity states in mice, rats, monkeys or humans were used as a context-specific input for building the reference network. The source studies collection included 10 studies comparing control and diet-induced obesity experimental groups and showing statistically significant differences in subject’s adiposity level between the two groups. The assays included gene expression, clinical chemistry and physiological data, measured in either WAT, PBMCs, or blood (Table 2). The experimental data were subjected to integrative statistical analysis, and the resulting set of variables differentiating lean from obese groups (aggregated FDR corrected *p* < 0.01) was used as seed nodes for building the reference network (*n* = 1,026, Supplemental Table 5). In addition, experimental data were correlated with data on expert’s knowledge-defined markers of key physiological processes, and features showing statistically significant correlations (|*r*| > 0.7) were added to the list of seed nodes for building the reference network (*m* = 75).

**Table 2:** Experimental datasets used to build the white adipose tissue health reference network

Title	Accession/Reference	Species	Tissue	Data type	Source
Dietary restriction of mice on a high-fat diet induces substrate efficiency and improves metabolic health	GSE27213	Mouse	Adipose (epididymal)	Transcriptomics, Physiology, Clinical chemistry	Bioclaims
Short-term, high-fat feeding-induced changes in white adipose tissue gene expression are highly predictive for long-term changes	GSE38337	Mouse	Adipose (epididymal)	Transcriptomics, physiology, clinical chemistry	Bioclaims
Early biomarkers identified in a rat model of a healthier phenotype based on early postnatal dietary intervention may predict the response to an obesogenic environment in adulthood	Torrens et al.	Rat	PBMC, adipose (retroperitoneal)	Transcriptomics (PBMC), qPCR (adipose), clinical chemistry	Bioclaims
n − 3 PUFAs in obese and non-obese volunteers	See Supplemental Data 2	Human	Blood	Clinical chemistry, physiology	Bioclaims
Short-term fatty acid intervention elicits differential gene expression responses in adipose tissue from lean and overweight men	E-TABM-377	Human	Adipose (subcutaneous)	Transcriptomics	External
Assessment of diet-induced obese rats as an obesity model by comparative functional genomics	GSE8700	Rat	Adipose (epididymal)	Transcriptomics	External
Diet and feeding condition induced gene expression in rat peripheral blood mononuclear cells	GSE14497	Rat	PBMC	Transcriptomics	External
Diabetes biomarker disease progression study in rat adipose tissue	GSE13268	Rat	Adipose (abdominal)	Transcriptomics	External
Time-course microarrays reveal early activation of the immune transcriptome and adipokine dysregulation leads to fibrosis in visceral adipose depots during diet-induced obesity	GSE39549	Mouse	Adipose (visceral)	Transcriptomics	External
Resveratrol improves adipose insulin signaling and reduces the inflammatory response in adipose tissue of rhesus monkeys on a high-fat, high-sugar diet	GSE50005	Macaca mulatta	Adipose (Subcutaneous)	Transcriptomics	External

### Prior knowledge-based molecular interaction network

To improve completeness of biological relations relevant to WAT health status, experimental findings were extended with prior knowledge of molecular interactions derived from public databases. The information included protein–protein interactions, protein–metabolite interactions, transcription factor targets, microRNA targets, pathway interactions and protein–drug interactions (Table 3, “Methods” section). The complete prior knowledge interaction network (27,667 nodes and 447,174 edges) was used as the molecular context for seed nodes derived from experimental data and correlation analysis.

**Table 3:** Number of nodes and edges in the complete knowledge-based network and the white adipose tissue reference network (WATRefNet), total and per tissue (i.e., blood, physiology, adipose and PBMC)

	Complete knowledge-based network	Total WATRefNet	Blood	Physiology	Adipose	PBMC
Nodes						
Gene/protein	14,488	4,361	23	0	4,234	104
Metabolite	12,729	2,349	18	0	2,241	90
Non-molecular	18	23	5	11	7	0
miRNA	432	64	0	0	64	0
Total	27,667	6,797	46	11	6,546	194
Edges						
DrugBank	9,494	504	0	0	504	0
KEGG	195,867	5,682	1	0	5,622	59
STITCH	76,269	6,973	47	0	6,691	235
STRING	155,971	17,910	46	0	17,745	119
TFe	1,929	259	1	0	258	0
WikiPathways	18,601	2,989	0	0	2,983	6
MirTarBase	3,597	265	0	0	265	0
Correlation	0	32	1	28	3	0
Total (merged)	447,174	32,171	95	28	31,645	403

Different resources for edges comprise different edge types (DrugBank: drug–target interactions, KEGG: manually curated metabolic and signaling pathways, STITCH: chemical–protein interactions, STRING: protein–protein interactions and associations, TFe: transcription factor–target interactions, WikiPathways: manually curated metabolic and signaling pathways, MirTarBase: manually curated miRNA–target interactions

### Construction of WAT health reference network

**Fig. 1:**
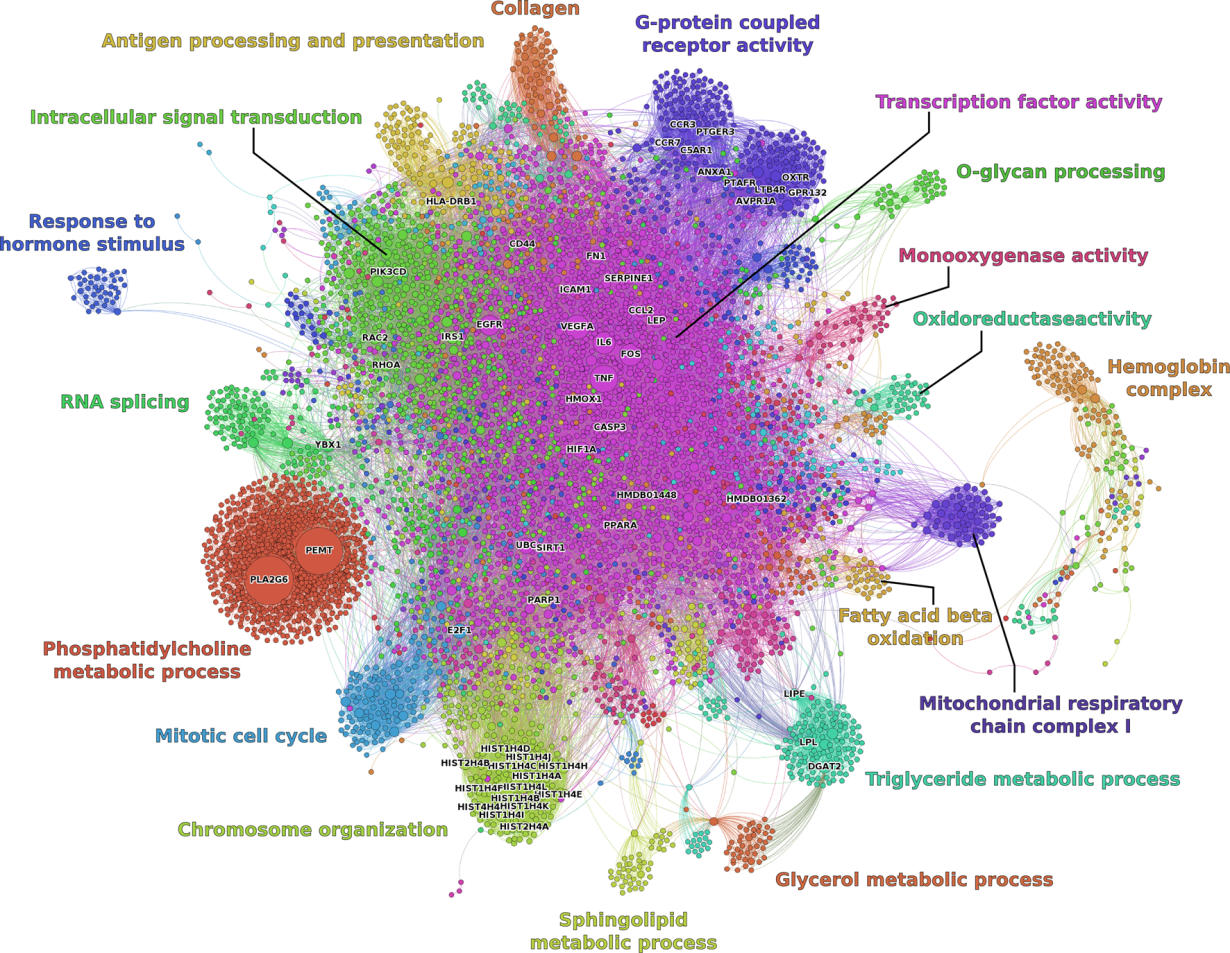
Visualization of the white adipose tissue health reference network. Nodes are colored by clustering based on network topology. Clusters are annotated with biological function based on GO overrepresentation analysis (“Methods”). Node size is scaled according to degree (number of interactions)

To construct the WATRefNet, the seed nodes derived from experimental data were integrated with molecular interaction context of the prior knowledge network. The subgraph based on the seed nodes was expanded by their first-order neighborhood, followed by a pruning step where all seed node neighbors that connected to only a single seed node were removed (“Methods” section). This resulted in the WATRefNet containing 6,797 nodes and 32,316 edges (Table 3; Fig. 1). The resulting network shows typical topology properties of biological networks (Barabási and Oltvai 2004; Albert 2005), such as scale-freeness (power law fit of degree distribution *R* ^2^ = 0.805) and hierarchical organization (power law fit of clustering coefficient distribution *R* ^2^ = 0.541) (Supplemental Table 1).

### Validation of WAT health reference network by enrichment with disease-associated gene sets

The WATRefNet was based on information originating from both human and animal model studies. To assess the relevance of the reference network for human disease, we have performed enrichment analysis of genes/proteins in the WATRefNet among known disease-associated genes. The disease-associated genes included 103 genes annotated as obesity-associated in the Gene2MeSH database (“Methods” section). In total, 70 disease genes were present in the reference network, representing a significant enrichment of reference network genes in this gene set (*p* = 1.09E−46). This finding supports the robustness of WATRefNet and its validity for assessing health effects in human intervention studies.

The reference network was also found to be enriched with known anti-obesity drug targets (Table 4, *p* = 7.46E−14), confirming that reference network represents a useful knowledge resource for finding molecular paths to be targeted by health interventions.

**Table 4:** Enrichment of the White Adipose Tissue reference network (WATRefNet) with different disease-relevant gene sets

	Obesity	ADT all depots	ADT visceral	ADT subcutaneous	ADT gonadal	Drug targets
Total genes	47,938	47,938	47,938	47,938	47,938	47,938
Total disease genes	103	1,208	0	77	1,148	54
AdipRefNet genes	4,194	4,194	4,194	4,194	4,194	4,194
AdipRefNet disease genes	70	475	0	35	454	27
Fisher exact test *p* value	2.59E−49	1.44E−190	1	2.02E−17	4.55E−183	4.60E−15

“Total genes” refers to total number of human genes in Entrez gene database. Obesity: Genes linked to MeSH term “Obesity”, ADT: differentially expressed genes in the anti-diabetic treatment study, Drug targets: anti-obesity drug targets from DrugBank

In addition, the WATRefNet was also found to be enriched with differentially expressed gene sets from an independent study comparing chow versus high-fat feeding conditions in LDLr−/− mice (Radonjic et al. 2013). Differentially expressed genes in either gonadal, visceral or subcutaneous fat depots were significantly enriched in the WATRefNet (475 out of 1,228 differentially expressed genes present in the reference network, Fisher exact test *p* = 5.75E−171, for enrichment in individual depots, see Table 4), confirming its robustness.

### Functional annotation of WAT health reference network

The WATRefNet can be clustered in 192 topological modules (Fig. 1; Supplemental Table 2). Functional annotation of these modules reveals that key biological categories determining health status of WAT involve: Transcription factor activity, Phosphatidylcholine metabolic process, Intracellular signal transduction, G protein-coupled receptor activity, Chromosome organization, Triglyceride metabolic process, Mitotic cell cycle, Antigen processing and presentation and RNA splicing. Interestingly, regulatory modules (with GO annotation transcription factor activity and intracellular signal transduction) are among the modules with the largest number of nodes and are central to the entire network. In contrast, modules annotated with mitochondrial processes, metabolism (e.g., Triglyceride metabolic cluster), immune process (e.g., Antigen processing and presentation cluster), cell division and structural remodeling are located at the periphery of the network and are connected to the central, regulatory part by several “bridging” nodes (Supplemental Table 3, “Methods” section).

For instance, LPL, DGAT2, LIPE, PNPLA2 and PNPLA3 are bridging nodes for the Triglyceride metabolic process cluster (cluster 96). The Antigen processing and presentation cluster (cluster 9) is linked through hsa-miR-16, multiple HLA class II histocompatibility antigens chains (HLA-DRB1, HLA-DRB5, HLA-DMB, HLA-DMA and CD74) and other immune response players like IRF5 or RNASEL. Leptin, Resistin and Adiponectin form the entry points into the Response to hormone stimulus cluster (cluster 21). These bridging molecules may be considered as mediators for the given biological functions, and targeting these molecules by intervention may result in profound effects on associated processes. In addition, bridging nodes that are not part of the functionally annotated modules, but are top-ranked molecules based on their betweenness centrality (i.e., KLF15 and WT1), may be interesting candidates for further research.

### Network signatures of physiological processes key to WAT health

**Fig. 2:**
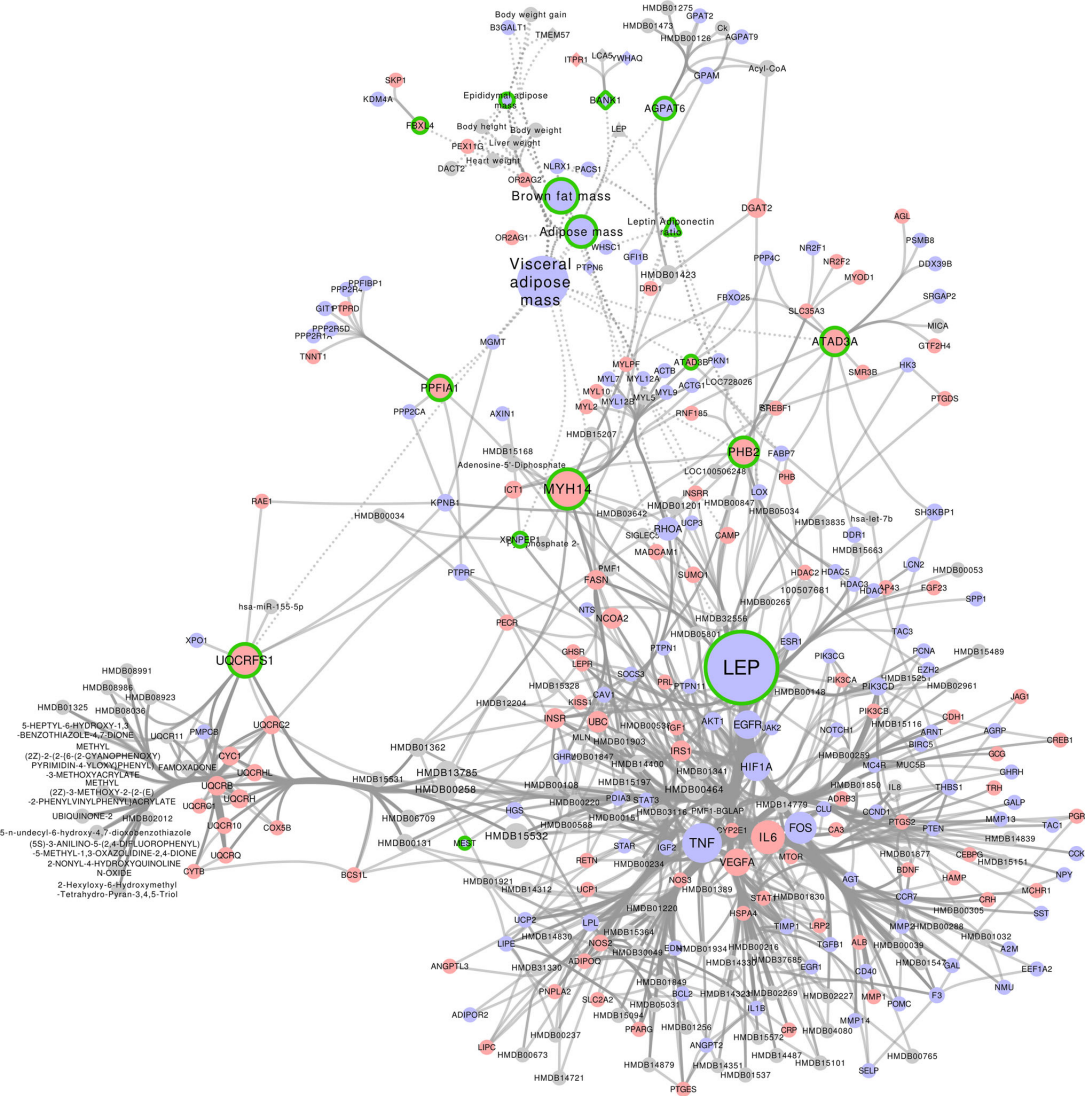
The network signature for process Adipose expandability. Nodes are colored according to the sign of the average fold-change across different studies (*blue* negative and *red* positive). Nodes with a green border are seed nodes (i.e., significant aggregated p-value and consistent fold-change across studies), and other nodes are neighbors of these seed nodes and included in the network to add biological context. Solid edges indicate knowledge-based molecular interactions; dotted lines indicate interactions based on correlations in the reference datasets

To identify parts of the WATRefNet that can be used as composite biomarker signatures for specific physiological processes key to WAT health, we have (1) extracted subnetworks constituting molecular neighborhood of the expert-defined markers and (2) pruned these subnetworks to include only molecules that show statistically significant changes in experimental data (aggregated FDR corrected *p* < 0.01) and their direct neighbors. This resulted in five process-specific network signatures, containing a prioritized list of key molecules that can together be used as robust indicators of the status of a given physiological process in intervention studies (Supplementary Table 4). Figure 2 shows a network visualization of the Adipose expandability signature, where the molecular interactions are visualized together with different relevant criteria, such as direction of gene expression, significance of differential expression and centrality of each marker in the network. As molecules constituting the network signatures are mechanistically linked to the physiological process of interest, their change upon intervention may suggest a cause–effect relationship between molecular changes and a health effect.

### Using network signatures for evaluating health effects of interventions

**Fig. 3:**
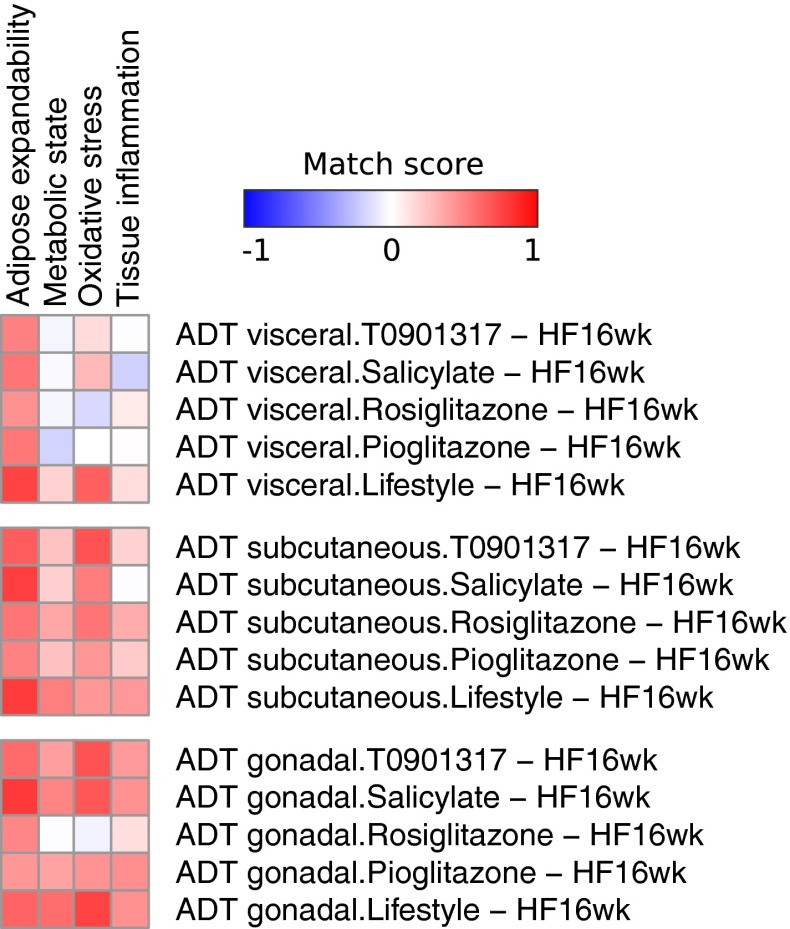
Overlay of intervention study (GEO Accession GSE57659) on the network signatures for specific processes related to white adipose tissue health. The heatmap shows the matching scores for each signature and intervention combination, where red indicates a positive score (positive “healthy” effect) and blue indicates a negative score (negative “disease” effect). Matching scores for the Oxidative capacity signature could not be calculated for any of the interventions due to lack of sufficient measurements of the markers in this signature

To assess the potential of the network signatures to be used as robust biomarkers mechanistically linked to physiological processes, which in turn determine WAT health, we have analyzed data of five intervention studies for their profile match with the network signatures. Gene expression changes in WAT of LDLr−/− mice upon one dietary and four drug interventions (Radonjic et al. 2013) were overlaid over the signatures, and the matching scores were calculated. The matching score was based on the correlation between fold-changes of differentially expressed genes in the network signature and the corresponding fold-changes resulting from the control versus intervention comparison (“Methods” section). A positive score represents a good correlation of the intervention with the reference expression, indicating that the intervention resulted in a healthy profile. A negative score indicates that the intervention resulted in an unhealthy profile. Figure 3 shows a heatmap of the matching scores for each signature and intervention combination. In line with previously reported ability of dietary lifestyle intervention to revert disease parameters (Radonjic et al. 2013), the matching scores indicate a healthy signature for this intervention for all network signatures. The signatures also reveal adipose depot-specific responses to the drug interventions. The response in gonadal and subcutaneous WAT results in a positive score, while the scores in visceral WAT are mixed and closer to zero indicating a weaker and/or less consistent effect of the intervention. The effect of salicylate intervention on Tissue inflammation signature results in particularly diverse matching scores in the three WAT depots (positive in gonadal, neutral in subcutaneous and negative in visceral), suggesting an interaction between drug mechanism of action and specific metabolic role of the three WAT depots. To test the statistical significance of difference between three depots, we have performed a one-way ANOVA test on scores for drug interventions (within each signature) comparing the three depots. In summary, significant difference among depots is observed for the Oxidative stress (*p* value 0.045), Metabolic state (*p* value 0.007) and Tissue inflammation (*p* value 0.01) network signatures. In contrast, the Adipose expandability signature is rather comparable among three depots (*p* value 0.49). In addition, in the gonadal depot, rosiglitazone intervention is markedly different than the other interventions. Although both rosiglitazone and pioglitazone act as PPARy agonists, they lead to different clinical outcomes (Deeg and Tan 2008), and differences in their specific signatures may help to elucidate the mechanisms responsible for this difference (Supplemental Table 6).

### Evaluation of predefined expert-based biomarkers in the context of WATRefNet and network signatures

Finally, we have evaluated novel insights emerging from the WATRefNet and network signatures, compared to previously known, expert-defined biomarkers of WAT health. We first investigated expert-defined markers for their relevance according to the data-driven approach (Table 1). Out of 75 expert-defined markers, 56 were measured in at least one of the experimental datasets and 23 of these showed consistent change across all datasets (consistent fold-change sign and aggregated FDR corrected *p* < 0.01). Thus, only one-third of expert-defined biomarkers are reconfirmed as eligible biomarkers by a purely data-driven approach. If we include additional markers based on their molecular context (i.e., being direct neighbor of at least 2 data-driven seed nodes), additional 24 expert-defined biomarkers can be included in the network as relevant, based on indirect association. The remaining nine expert-defined biomarkers which have been measured but do not show either consistent change in the data or are in known molecular neighborhood of the seed nodes are—according to our analysis—of questionable relevance as biomarkers of adiposity state (Table 1).

### Added value of network signatures as biomarkers of WAT health

The expert-defined biomarkers are per definition included in the network signatures for WAT health. We next asked whether other, newly discovered biomarkers within these signatures may in fact offer a more reliable picture of the adipose state than the expert-defined ones. Inspection of individual nodes in the network signatures identifies novel molecules, which outperform expert-based ones (Supplementary Table 4). For instance, in the “Oxidative stress” network signature, there are 20 novel markers with lower aggregated *p* value then expert-defined biomarkers LPL and LDHA. Investigation of biological functions of these 20 markers as a group suggests that changes in extracellular matrix organization and leukocyte migration may be a good indicator of oxidative stress in WAT. In turn, the “Metabolic state” signature confirms the relevance of expert-defined biomarkers ACACA, FASN and PDK1 (all within top four markers based on aggregated *p* value). Interestingly, the next four top-ranked molecules are newly discovered, and all involved in cholesterol biosynthesis (G6PD, CYP51A1, PMVK and FDPS). In addition to using network signatures as a biomarker of health, they may be explored in a multitude of ways and hint to underlying mechanisms. This may lead to development of mechanism-driven, noninvasive assays for assessment of WAT health (e.g., leukocyte status for oxidative stress or plasma cholesterol levels as indicator of WAT metabolic status).

## Discussion

We have constructed a WATRefNet as a resource for discovery and prioritization of mechanistically supported biomarkers for health benefits of food compounds. The presented work provides a step forward in understanding and quantifying health by shifting the focus from single, correlation-based biomarkers toward composite, mechanistically supported biomarker signatures. Also, the reference network concept can be used as a solution for integration and mining of context-specific multi-layered datasets and prior knowledge on biological interactions for different health-related processes. This results in a sustainable knowledge resource for assessment of health status and effects of health-improving interventions.

### Understanding and quantifying health

Achieving and maintaining optimal health remains a challenging task due to complexity of the involved factors. For instance, drug interventions are typically designed to strongly target a single-defined molecule—a strategy that promotes efficacy but increases the risk of adverse effects. In cases where homeostasis is still not fully disturbed, dietary and other lifestyle interventions may be particularly suitable to improve health or reverse a course of disease due to their mild effects on a broad collection of different mechanisms, often across different organs. The proposed network-based data analysis strategy facilitates underpinning of system-wide effects of dietary and lifestyle interventions, allowing comprehensive mapping of factors relevant for specific health area—in this case WAT health. As the resulting reference network is built in a context of diet-induced change in adiposity state, it provides a “healthy” versus “less healthy” WAT-specific signature. As such, the WATRefNet is a resource for extraction of relevant relationships and features of interest (e.g., mechanisms, biomarkers and intervention targets; Fig. 1, Supplemental Table 2), but also allows quantification of a “match” of molecular changes induced by interventions to the one of the “healthy” signature. The possibility to score the health effects of interventions makes such context-specific reference networks a valuable tool for quantitative assessment of health effects of interventions, as demonstrated by the example of five anti-diabetic interventions (Fig. 3).

### Physiologically relevant biomarker signatures

The primary goal of the FP7 BIOCLAIMS project is to identify and characterize robust, nutrigenomic-based biomarkers predictive of a healthy metabolic phenotype facing perturbation of homeostasis. In particular, the focus is on developing biomarkers for mapping the intrinsic effects of food components, which together might provide scientific evidence to help support future health claims on food. In the context of FP7 BIOCLAIMS project goals, and within a broader scope of supporting approval of food health effect claims by the European Food Standards Agency (EFSA 2006), we have here exploited the reference network concept for extraction of robust biomarker signatures for assessment of health status and health benefits of interventions. Its validation in practical developments in the food/health economic sector should be tested in humans under a number of different conditions.

The key requirement for biomarker definition within our approach is that it reflects a physiologically relevant process, crucial for determining health state of a system under investigation. As the definition of health implements a view of optimally functioning human physiology as the ability to adequately adapt to one’s environment (Kitano et al. 2004; Stelling et al. 2004), the processes were selected based on their relevance for maintaining systems flexibility or robustness. This flexibility can be established and maintained at all levels of the system, e.g., whole body, organ, cellular and subcellular. The joint effort of BIOCLAIMS consortium members resulted in definition of key “robustness” processes of WAT health, namely Adipose expandability, Oxidative capacity, Metabolic state, Oxidative stress and Tissue inflammation. These processes are therefore used here as a basis for conveying changes observed in biomarker signatures to WAT health benefits.

Another innovative aspect of our biomarker definition is the inclusion of mechanism-based selection criteria to complement correlation statistics variable selection methods. This is achieved in the first instance by (1) requirement of a biomarker to be associated with one of predefined physiologically relevant processes and (2) including the mechanistic context, i.e., molecular interaction neighborhood of a given biomarker. This approach increases the chance that a selected biomarker is not only statistically significant, but also biologically relevant for the health endpoint of interest. In addition, we extract a composite biomarker signature instead of focusing on isolated entities, which likely further increases the robustness of biomarker selection. This resulted in identification of five mechanistically supported biomarker signatures, functionally and statistically linked to the physiological processes determining robustness of WAT and therefore to the WAT health (Supplemental Table 4). Importantly, based on these signatures, a matching score can be calculated to quantify health effects of interventions (Fig. 3).

Each individual biomarker within a signature can be prioritized based on different relevance criteria, e.g., centrality (importance) in the network, magnitude of changes in experimental data, annotation, the ability of the molecule to be secreted and cellular localization. (Supplemental Table 4). While such ranking of individual markers allows flexibility in designing scope of validation experiments (e.g., if only a limited number of biomarkers can be measured in a clinical study), the availability of the complete molecular context facilitates exploration of data and knowledge to generate novel hypotheses and drive development of improved interventions (Kelder et al. 2010).

An important characteristic of a biomarker is its feasibility to be measured in human clinical studies. Therefore, biomarkers which are indicative of tissue health status but which can be measured by noninvasive methods (e.g., in plasma or urine samples) are of great practical value. The WATRefNet can be exploited for this purpose for identification of markers derived from PBMC or plasma samples and which are located in the network neighborhood of five biomarker signatures. This concept would ideally be expanded to include other types of noninvasive assays or measurement platforms, such as molecular imaging or metabolomics and (epi)genetics in accessible tissues or body fluids.

### Reference network as a sustainable knowledge resource for assessment of health status and effects of interventions

To meet the challenge of comprehending complex biological relations relevant for health, we introduce here the concept of reference networks as a mean for multi-level mapping of systems components and interactions between them. We use reference networks as a platform for integration and mining of biological interactions derived from public resources and context-specific experimental data. This enables understanding of the high-level organization of processes that are required for maintenance of WAT health, bridging information at the level of organs or tissue, to physiological processes to molecular interactions. Specifically, the reference network platform facilitates the following functionalities.

#### Integration of diverse and abundant data

To warrant comprehensiveness and robustness of the WATRefNet, we have integrated biological information originating from multiple experimental evidences (i.e., 10 studies, five species, three assay platforms and four tissues), multiple prior knowledge resources of molecular interactions (7 public knowledge databases) and domain knowledge within BIOCLAIMS.

#### Mining of features and relations

Relying on a graph-based theory and prior knowledge integration, network biology facilitates extraction of features (biomarkers and intervention targets) and relations (mechanisms, codependencies and causality), which are not only statistically significant but also biologically relevant. Involving biology context into data mining is especially relevant in nutritional research, as typically mild and broad effects can be overlooked by stringent pure statistical methods (Ideker et al. 2011). In addition, accounting for molecular context (e.g., pathway cross talk) provides valuable information for understanding mechanisms underlying health status and intervention effects and is important to consider when designing or benchmarking novel interventions. Good examples of this concept are Leptin and Adiponectin, known indicators of WAT health status (Guerre-Millo 2004). The WATRefNet confirms an important role for these molecules, as Leptin, Adiponectin and Resistin represent key nodes linking peripheral Response to hormone stimulus cluster to the central regulatory cluster of the WATRefNet. Similarly, we discover PNPLA2 and PNPLA3, for which genetic variations have previously been associated with obesity (Johansson et al. 2008) and non-alcoholic fatty liver disease (Romeo et al. 2008), as the key nodes bridging Triglyceride metabolic process cluster with central regulatory mechanisms. In addition, we found Hsa-miR-16 as key node linking the antigen processing and presentation cluster to the reference network. This is a relatively unknown miRNA that has been mostly associated with various cancers (Calin et al. 2008), and our results indicate relevance in context of the inflammatory process in WAT health as well. Finally, KLF15 and WT1 emerge from our analysis as bridging nodes, top ranked according to betweenness centrality, but not constituting functionally annotated modules. KLF15 has previously been associated with glucose-induced insulin secretion in adipocytes, confirming its relevance for adiposity and WAT health (Nagare et al. 2011). In turn, WT1 is involved in development and tumorigenesis (Toska and Roberts 2014), and it may be an attractive novel candidate for further analysis in the context of WAT health.

#### Organizing and storing of knowledge (instead of data) in form of relations

Instead of dispersed data (i.e., dozens of data files containing tens of thousands data points, scattered across six BIOCLAIMS institutes and different public repositories, in different formats and annotations), we have generated a traceable, transparent, annotated and accessible resource of knowledge about WAT health, documented in a form of entities and their relations. This resource is available in a computer readable formats (Supplementary Dataset 1), ready for efficient mining for different aspects of interest [i.e., by applying network-based path finding (Kelder et al. 2011)], prioritization or clustering algorithms (Warde-Farley et al. 2010), cross-reference with other network signatures (Wang et al. 2012), or querying specific parts of interest], as information layers and relations can be flexibly extracted.

#### Sustainability of generated data and knowledge

The reference networks concept provides a sustainable solution for use and reuse of data that has been incorporated into the network. Reference networks can be stored in a dedicated infrastructure (i.e., network library), allowing research question-driven mining at any future time point (NDEx 2014). In addition, multiple reference networks can be readily integrated, for instance, to address several related health aspects (Shannon et al. 2003). Importantly, network architecture allows expansion and refinement of the existing model upon incorporation of novel information, meaning that if more or better evidence becomes available, the network model will improve.

Together, these properties imply that the value of a reference network will increase as the knowledge increases, which will in turn facilitate novel discovery, hypothesis forming and validation—therefore reinforcing each other. This concept holds a promise of growth in scientific value, where newly initiated research will optimally build upon prior experience—our own and that of others. Within the FP7 BIOCLAIMS project, we have demonstrated the viability of this concept by integration of data and knowledge across six different European institutes, publicly available experimental datasets and knowledge on molecular interactions. The obtained biomarker signatures for five key physiological processes determining health of WAT are already being used as an input for analysis in other related projects focusing on metabolic health (Bobeldijk et al. 2014).

Application of this and similar data management models allows cross talk between different (parts of) projects and will therefore generate an enhanced output. This helps to improve our understanding of the causes and mechanisms underlying health and disease and obtain insight in treatment effectiveness. Hereby, we demonstrate the importance of availability of open data and joint data/knowledge mining efforts for successful implementation of such models.

### Limitations and future perspectives

The undertaken approach is intrinsically limited by availability and quality of data used for the analysis. Despite a fairly comprehensive mapping of relations relevant for WAT health, the bias in, e.g., data and/or tissue types, is unavoidable. For instance, transcriptome data availability overrules other omics assay platforms, and WAT transcriptome data obtained from rodent models are more abundant than such data from human studies. In addition, it has been shown that different fat depots, such as subcutaneous, visceral or gonadal fat, have different roles and metabolic properties (Caesar et al. 2010), and their translational value is also questionable due to different spatial distribution in human and animal models. Ideally, these differences would be accounted for by defining adipose depot-specific layers in the WATRefNet. Currently, the available depot-specific data are insufficient to properly address this aspect. Apart from limitation in experimental data, sources of prior knowledge of molecular interactions have different reliability (Von Mering et al. 2002). For instance, protein–protein interactions derived from in-depth mechanistic protein interaction analysis will likely yield less false-positive findings than high-throughput screens, such as yeast two-hybrid system. To account for these different confidence levels, elements in the network may be assigned different weights, based on reliability of the evidence source. Another approach to correct for relations that could potentially be found by chance—especially when used as a series of associations constituting the analysis workflow—is to compare the resulting network to the randomized network starting form an equivalent set of nodes (Aittokallio and Schwikowski 2006). In addition, to improve sensitivity of the network signature scores, nodes could be weighted based on their biological/mechanistic relevance. One possible method of weighting individual parameters would be to manually define higher weights for established physiological readout parameters. A more unbiased model of assigning relevance weights could be made using network topology features—assuming that our knowledge on underlying molecular network is sufficiently complete. This is an emerging field, and, to the best of our knowledge, this approach has not yet been extensively benchmarked to be routinely included in our current analysis. We envision that resources and models (including signatures provided in this work) will be refined in parallel with growth of available knowledge, experimental evidence and network models. Ultimately, the optimal solution to refinement of network models is to incorporate statistically sound and biologically supported checkpoints at each workflow step, and where possible, experimentally validate anticipated relations. The prerequisite for successful reference network analysis is a deep understanding of both technological requirements and the biological context, allowing careful selection of data and knowledge resources. To achieve this, a joint effort and multi-disciplinary approach are a pro, possibly involving network scientists, mathematicians, statisticians, bioinformaticians, biologists, nutritionists, medical doctors and policy makers. There is a need to establish a bridge between the prioritized biomarkers and the adaptation of the practical requirements currently established for biomarkers and risk factors in the context of health claims made on food in Europe (EFSA Panel on Dietetic Products N and A (NDA) 2011). The application of the present method may provide important information to show the biological plausibility of specific effects of foods on health, which acceptability under the current European Regulation (EC) Nº 1924/2006 on health claims made on food, will be difficult to be based only on a few systematic well-controlled human intervention studies.

Although the method has been developed focusing on diet-induced changes in WAT health, the concept is generic and applicable to the broader area of (metabolic) health and disease. This provides opportunities for supporting a wide range of applications, such as design of improved food or drug interventions, substantiation of health claims of food products, efficacy/safety analysis of drug therapies and pinpointing health effects of combination therapies. Finally, the relevant implications of reference network approach are a potential to apply it in a “*n* = 1” approach, leading to extraction of person-specific health signatures and quantification of person-specific health effects compared to, for example, a population pool (Chen et al. 2012). This will facilitate development of personalized interventions and more efficient, subgroup-specific intervention protocols in clinical trials. Together, the health reference network concept as a sustainable knowledge resource and associated robust health biomarker signatures open numerous new avenues for assessing and quantifying health and the effect of interventions on thereof.

## Methods

### Collection and formatting of experimental data

Experimental data across 10 studies (Table 2) were collected from public repositories [GEO: GSE27213 (Duivenvoorde et al. 2011), GSE8700 (Li et al. 2008), GSE14497 (Caimari et al. 2010), GSE13268 (Xue et al. 2011), GSE39549, GSE38337 (Voigt et al. 2013), GSE50005 (Jimenez-Gomez et al. 2013), ArrayExpress: E-TABM-377 (Van Erk et al. 2008)] and BIOCLAIMS consortium members [(Torrens et al. 2014) and Supplemental Data 2]. All data were annotated to unified identifiers (Entrez Gene for genes/proteins, HMDB for metabolites) where possible, and names of physiological parameters were normalized to be consistent across different studies and match the expert’s defined markers (Table 1).

### Within-study statistics

For each dataset, a control (healthy and lean) and disease (high-fat diet and obese) group was defined, and a groupwise comparison was performed between these groups within each dataset. For transcriptomics data, the R package limma (Smyth 2004) was used to test for differential expression between the groups. For non-transcriptomics markers, a Student’s *t* test was applied.

### Integration of statistics across studies

To combine the within-study statistics across the different datasets, an aggregated *p* was calculated for each marker using Fisher’s method (Mosteller and Fisher 1948). The resulting *p* values were corrected for multiple testing using the Benjamini–Hochberg method for controlling the FDR (Benjamini and Hochberg 1995).

### Correlation analysis

Each expert’s defined marker was correlated within each study with all other markers measured in that study. Spearman’s rank correlation coefficient was calculated for all subjects of the healthy and disease groups, and correlations with an absolute correlation coefficient above 0.7 were included in construction of the reference network.

### Selection of seed nodes

A set of seed nodes relevant for WAT health was defined based on expert’s defined markers (Table 1) and markers derived from the analysis of the datasets. Seed nodes based on data analysis were selected based on the following criteria:

1. Aggregated *p* < 0.01.
2. Fold-change between health and disease group should be equal for all studies where the changes for the marker are significant according to criteria 1, i.e., the marker should change in the same direction consistently across all datasets.

Seed nodes based on correlations are included by selecting all markers that correlate with one or more of the expert’s defined markers (absolute correlation coefficient >0.7).

### Building the prior knowledge-based molecular interaction network

An integrated molecular interaction network was built by integrating the following network resources:

1. Protein–protein functional interactions from STRING (version 9.05) (Jensen et al. 2009), including all interactions with score >0.4 and excluding NLP-derived interactions.
2. Protein–metabolite interactions from STITCH (version 3.1) (Kuhn et al. 2010), including all interactions with score >0.4.
3. Transcription factor targets from the Transcription Factor Encyclopedia (version 2014-02-13) (Yusuf et al. 2012).
4. WikiPathways human pathways (Analysis collection, version 2013-08-14) (Kelder et al. 2012).
5. Gene–miRNA interactions from MirTarBase (from CyTargetLinker collection, version 2012-10-12) (Kutmon et al. 2013; Hsu et al. 2011).
6. Protein–drug interactions from DrugBank (from CyTargetLinker collection, version 2012-10-12) (Kutmon et al. 2013; Wishart et al. 2006).

Nodes were annotated to Entrez Gene (for genes/proteins) and HMDB (for metabolites) whenever possible using the BridgeDb library (Van Iersel et al. 2010). If no mapping to these identifier systems could be found, the original annotation was retained (e.g., for miRNAs). Edges were considered undirected, and edges were merged whenever an interaction was defined in multiple resources. Information regarding datasource of origin for each edge was retained in the network and is available in the network as edge attributes (Supplementary Data 1).

### Construction of WAT health reference network

The adipose health reference network (WATRefNet) was constructed from the combination of the integrated knowledge-based molecular interaction network and the significant correlations, resulting from the integrated data analysis. The WATRefNet was extracted from this network by taking the subgraph defined by the seed nodes and their first-order neighborhood. After extracting the subgraph, a pruning step was performed in which all seed node neighbors that connected to only a single seed node were removed. This step was performed to ensure that nodes added based on the neighborhood to the seed nodes provide relevant biological context and contribute to connecting different seed nodes. Topology parameters of the WATRefNet were calculated using the Network Analyzer App in Cytoscape (Assenov et al. 2008).

### Topology-based clustering and functional annotation

The WATRefNet was clustered into topological modules using the WalkTrap community detection algorithm (Pons and Latapy 2005). For each resulting cluster, overrepresentation analysis of the genes in the cluster with Gene Ontology terms from the Biological Process, Molecular Function and Cellular Location ontologies was performed using the GO enrichment Analysis in the WGCNA R library (Langfelder and Horvath 2008). All significantly overrepresented GO terms (*p* < 0.0001) were selected and listed in Supplemental Table 2.

### Validation with external gene sets

To validate the relevance of the WATRefNet, it was tested for enrichment with different external list of genes relevant to adipose health. Enrichment was tested using the Fisher exact test, with as reference set the complete collection of human Entrez Genes. The set of genes related to obesity was queried from the Gene2MeSH tool (Ade et al. 2007), resulting in 103 genes associated with the MeSH term “Obesity.” The sets of differentially expressed genes (FDR corrected *p* < 0.05) in an independent transcriptomics dataset were defined based on transcriptomics measurements in different adipose depots (subcutaneous, visceral and gonadal) for the comparison of chow versus high-fat feeding conditions in LDLr−/− mice (Radonjic et al. 2013) (GEO Accession GSE57659). Although we are of the opinion that nutritional studies should make use of comparative purified diets to dissect effects of diets (Hoevenaars et al. 2012), we think that inclusion of a study that uses chow as comparator is warranted in this case, because the analysis focuses on contrast rather than on specific effects of a diet. The analysis resulted in 1,228 differentially expressed unique genes across all depots (Visceral: 2, Subcutaneous: 79 and Gonadal: 1,167). The set of 55 anti-obesity drug targets was obtained from DrugBank (Wishart et al. 2006).

### Identification of nodes bridging peripheral and central network clusters

The two central clusters in the network with GO annotation transcription factor activity and intracellular signal transduction were grouped and collapsed into singular nodes representing all module members. The module node retains all edges of its members. After collapsing the clusters, the betweenness centrality was calculated using the Network Analyzer App in Cytoscape (Assenov et al. 2008). The betweenness centrality was calculated based on all shortest paths in an undirected, unweighted network. This allows for the case that nodes can have the same betweenness. The betweenness centrality was normalized based on the number of nodes: 1/(n−1) × (n−2) and independent from the betweenness calculated, allowing for having nodes of equal betweenness even after normalization. Nodes with a betweenness centrality larger the 90 % quantile (2.86E−4) for each module were identified as bridging nodes.

### Extraction of process-specific network signatures

A network signature was extracted from the WATRefNet for each of the five physiological processes (Table 1). First, a subgraph was extracted based on first-order neighborhood of all expert’s defined markers associated with the process. Next, this subgraph was pruned by excluding all nodes that are not direct neighbors of a marker that was part of the data-driven seed nodes (shows statistically significant changes in the experimental data, aggregated FDR corrected *p* < 0.01).

### Matching network signatures with interventions

Signatures were matched to gene expression changes in WAT of LDLr^−/−^ mice upon one dietary and four drug interventions (Radonjic et al. 2013) to assess the health effect of the interventions. A matching score was calculated for each signature and intervention, defined as the Spearman correlation of fold-changes of the significantly changed markers in the signature and the fold-changes resulting from the intervention. Correlations were calculated only if data for at least three significantly changed markers were available for both the signature and the intervention dataset.

### Network analysis and visualization

Network analysis was performed in igraph (Csárdi and Nepusz 2006) and Cytoscape (Shannon et al. 2003). Network visualizations were performed in Gephi (Gephi et al. 2014) (Fig. 1) and Cytoscape (Fig. 2). The WATRefNet is available in computer readable format as RData file and Cytoscape session file (Supplemental Dataset 1).

## Electronic supplementary material

Below is the link to the electronic supplementary material.

Supplementary material 1 (ZIP 5046 kb)

Supplementary material 2 (DOCX 19 kb)

Supplementary material 3 (XLS 1191 kb)

Supplementary material 4 (XLS 95 kb)

Supplementary material 5 (XLS 371 kb)

Supplementary material 6 (XLS 3479 kb)

Supplementary material 7 (XLS 2018 kb)

Supplementary material 8 (XLS 25 kb)
