# Supplementary material for: White adipose tissue reference network: a knowledge resource for exploring health-relevant relations
Source: Genes Nutr. 2014 Dec 3;10(1):439. doi: 10.1007/s12263-014-0439-x (PMC4252261; doi:10.1007/s12263-014-0439-x)
Supplement: Supplementary file 2 — Supplementary material 2 (DOCX 19 kb) [file 12263_2014_439_MOESM2_ESM.docx]

**Methods**: 150 healthy subjects with BMI 25-40 kg/m^2^ aged 25-65 (mean 47) yrs. were included into the double-blind randomized EU BIOCLAIMS trial. Participants were on isocaloric diet, when 50 obese patients were put on low caloric diet. Patients were supplemented with capsules either with 3x600mg/day DHA:EPA (5:1) (EPAXTG , Norway) or with placebo for three months. Compliance was confirmed by mass spectrometry of n-3 PUFA content of erythrocyte phospholipid content and serum (USoton). Blood concentrations of markers of inflammation (hsCRP, IL-6, sE-Selectin, s-VCAM-1, sPECAM-1, MCP-1), and adipokines (leptin, adiponectin, resistin, visfatin, VEGF), as well as antioxidative potential markers (FRAP, glutathione peroxidase, glutatione reductase), and osteocalcin (the calcium regulating small gla-protein, which undercarboxylated in circulating form has been implicated as a novel hormone and positive regulator of glucose and adiponectin homeostasis) from blood samples obtained in the fasting state before and after supplementation were determined. Modification of LDL (metabolomics) was performed in cooperation with UWar (LC-MS/MS).
